# Sacrocolpopexy: The Way I Do It

**DOI:** 10.1007/s00192-024-05922-0

**Published:** 2024-10-15

**Authors:** Usama Shahid, Zhouran Chen, Christopher Maher

**Affiliations:** 1 Royal Brisbane and Women’s Hospital, Brisbane, Australia; 2 James Cook University, Brisbane City, QLD 4000 Australia; 3 St George Hospital, Sydney, Australia; 4 Royal Brisbane and Women’s and Wesley Hospitals, University of Queensland, Brisbane, Australia

**Keywords:** Sacrocolpopexy, Mesh, Exposure, Prolapse, Vault, Apical

## Abstract

**Introduction and Hypothesis:**

Sacrocolpopexy (SCP) is an established surgical procedure for apical vaginal vault prolapse. There remains significant variation amongst surgeons in both the surgical steps and concomitant surgeries utilised when undertaking an SCP.

**Methods:**

This review article is aimed at summarising the evidence and providing a detailed update of SCP in modern practice, reviewing contemporary evidence behind its indications, efficacy, outcomes, surgical steps, and complications.

**Results:**

Sacrocolpopexy remains the gold standard for post-hysterectomy apical prolapse based on good long-term outcomes, patient satisfaction and low complication rates. SCP with concomitant total hysterectomy is not recommended owing to high rates of mesh exposure. The laparoscopic approach remains the preferred option in terms of low morbidity, quicker recovery and lower cost than alternative access options. For optimal outcomes an SCP should be performed with monofilament mesh, using absorbable sutures and with a paravaginal repair for cystocele.

**Conclusions:**

Although SCP has become increasingly utilised for apical prolapse, its established efficacy regarding anatomical outcomes, patient satisfaction, and complications is in the context of post-hysterectomy prolapse. SCP with concomitant total hysterectomy has higher rates of mesh exposure. The efficacy and safety of SCP with sub-total hysterectomy or hysteropexy have not been clearly established and require further assessment through well-designed, rigorous randomised controlled trials.

## Introduction

Behind every medical innovation there lies the holistic and somewhat elusive goal of achieving “perfect” patient outcomes. This necessitates the implementation of gradual but continual improvements and the surgical procedure of a sacrocolpopexy (SCP) is no exception. Although first introduced in 1962 by Dr Fredrick Lane [1], SCP has advanced significantly regarding its outcomes, procedural steps, concomitant surgeries, and risk profile. In addition, although Dr Lane first described SCP in a four-patient case series, the procedure now accounts for 3.3% of all prolapse repairs undertaken in high-income countries [2]. Despite these developments, there remains significant variation amongst surgeons in both the surgical steps and concomitant surgeries utilised when undertaking an SCP. Usually, this attests to a lack of consensus (or evidence) on the matter. Thus, this article is aimed at providing a detailed update on SCP in modern practice, reviewing contemporary evidence behind its indications, efficacy, outcomes, surgical steps, and complications.

## Outcomes

### Efficacy: Is SCP Still the Gold Standard for Apical Prolapse Repair?

There is a growing body of evidence that suggests that in order to facilitate the durability of a prolapse repair, adequate vaginal apical support is required [3, 4]. Transvaginal suspensory approaches include sacrospinous ligament fixation (SSLF), McCall’s culdoplasty, uterosacral ligament suspension (USLS), Manchester repair, and vaginal mesh surgeries, whereas the obliterative pathway is namely a colpocleisis. Abdominally, the apex of the vagina can be suspended through an SCP or a USLS.

The most robust meta-analysis to date remains the 2023 Cochrane review entitled “Surgery for women with apical vaginal prolapse” [4]. The review identified seven randomised controlled trials (RCTs) comparing SCP with vaginal apical support procedures. The trials included had a total of 613 women, with follow-up ranging from 6 months to 4 years post-operatively; 83% of all participants included across the seven trials underwent an SCP for posthysterectomy prolapse. Of the trials included, 4 compared SCP with SSLF [5–8], 2 trials compared SCP with USLS [9, 10], 1 trial compared SCP with transvaginal mesh repair [10], whereas the final trial compared SCP with USLS with mesh augmentation [11]. The review found that awareness of prolapse (RR 2.31, 95% CI 1.27 to 4.21), recurrent prolapse on examination (RR 2.33, 95% CI 1.34 to 4.04), and repeat surgery for prolapse (RR 1.87, 95% CI 1.32 to 2.65) were all more common after vaginal procedures for apical vault prolapse compared with SCP. In addition, stress urinary incontinence (SUI) was more common after vaginal procedures than SCP (RR 1.86, 95% CI 1.17 to 2.94). The International Continence Society (ICS) in the seventh edition of their International Consultation on Incontinence (ICI) textbook (2023) included these findings as grade A evidence (Oxford Grading System) [12]. In addition, the ICI summarised 45 prospective and retrospective case series involving 5,584 patients undergoing an SCP. A high mean success rate of 93% (range 60–100%) was reported over a short- to mid-term follow-up period (Table 1). Similarly, there was a low mean re-operation rate of 7% and a mean re-operation for prolapse rate of only 3%.

**Table 1:** Outcomes for laparoscopic sacrocolpopexy studies with at least 12 months’ follow-up [12]

Reference	*n* (mesh)	Success rate	Follow-up (months)	Total reoperation rate	Reoperation rate, recurrence	Reoperation rate, complication	Vaginal mesh exposure	Spondylo-discitis	De novo dyspareunia
Cheret et al. [13]	44	43/44	18	0/44	0/44	0/44	0/44	0/44	0/44
Cosson et al. [14]	83	78/83	11	2/83	1/83	1/83	1/83	0/83	MD
Antiphon et al. [15]	108	75/100	16	10/108	5/108	0/108	0/108	1/108	MD
Gadonneix et al. [16]	46	38/46	24	0/46	0/46	0/46	0/46	0/46	MD
Higgs et al. [17]	103	39/66	60	15/103	11/103	4/103	6/103	0/103	MD
Ross et al [18]	51	48/51	60	10/51	3/51	4/51	4/51	0/51	4/51
Rozet et al. [19]	363	348/363	14	13/363	7/363	6/363	3/363	1/363	MD
Paraiso et al. [20]	56	MD	13	3/56	1/56	2/56	2/56	0/56	MD
Rivoire et al. [21]	114	100/114	33	14/114	7/114	7/114	7/114	1/114	0/114
Agarwala et al. [22]	74	74/74	24	2/74	0/74	2/74	1/74	MD	1/74
Stepanian et al. [23]	402	380/402	12	14/402	0/402	11/402	5/402	0/402	4/402
Misraï et al. [24]	43	37/43	48	3/43	1/43	2/43	2/43	0/43	MD
North et al. [25]	22	22/22	27.5	1/22	0/22	1/22	1/22	0/22	0/12
Claerhout et al. [26]	132	127/132	12	9/132	0/132	9/132	6/132	0/132	10/53
Deprest et al. [27]	65	43/65	33	7/65	0/65	7/65	7/65	0/65	MD
Sarlos et al. [28]	101	98/101	12	4/101	1/101	1/101	1/101	0/101	1/101
Granese et al. [29]	138	131/138	43	1/138	0/138	0/138	0/138	0/138	2/138
Akladios et al. [30]	48	46/48	16	8/48	0/48	2/48	1/48	0/48	MD
Sabbagh et al. [31]	186	122/132	60	**8/186**	2/186	6/186	5/132	0/132	9/170
Paraiso et al. [20]	29	21/23	12	0/29	0/29	0/29	0/29	0/29	MD
Sergent et al. [32]	124	103/116	34	10/124	MD	3/124	4/116	1/124	1/85
Price et al. [33]	84	84/84	24	7/84	4/84	3/84	5/84	0/84	MD
Maher et al. [11]	53	41/53	24	3/53	0/53	1/53	1/53	0/53	MD
Perez et al. [34]	94	88/94	12	0/94	0/94	0/94	3/94	0/94	2/94
Freeman et al. [35]	25	24/25	12	1/25	1/25	0/25	0/25	0/25	MD
Sarlos et al. [36]	85	57/68	60	5/85	3/85	2/85	2/85	0/85	MD
Costantini et al. [37]	60	60/60	41	3/60	0/60	3/60	3/60	0/60	1/60
Kenton et al. [38]	33	MD	12	3/33	0/33	3/33	0/33	0/33	MD
Chen and Hua [39]	102	102/113	24	MD	MD	MD	1/113	MD	MD
Vandendriessche et al. [40]	464	MD	54	49/391	20/391	11/391	11/391	MD	MD
Zhang et al. [41]	204	192/195	12	MD	MD	MD	1/204	MD	MD
Coolen et al. [42]	37	31/37	12	7/36	4/36	0/36	0/36	0/36	MD
Cormio et al. [43]	21	20/21	17	1/21	MD	1/21	0/21	0/21	MD
Vidal et al. [44]	72	71/72	18	7/72	0/72	0/72	0/72	0/72	2/72
Balsamo et al. [45]	73 (polypropylene)	67/73	94	0/73	0/73	0/73	1/73	0/73	MD
	63 (polyvinylidene fluoride mesh)	55/63	25.6	0/63	0/63	0/63	2/63	0/63	MD
Orhan et al. [46]	96 Gynae	83/96	48	11/96	6/96	0/96	2/96	0/96	MD
	94 Urology	82/94	48	10/94	5/94	0/94	2/94	0/94	MD
André et al. [47]	63	62/63	18	MD	MD	MD	0		2/63
Wagner et al. [48]	82	53/75	60	7/75	4/75	3/75	3/75	0/75	MD
Ozerkan et al. [49]	22	19/21	21	0/21	0/21	0/21	0/21	0/21	MD
Tibi et al. [50]	70	63/66	12	MD	MD	1/70	0/70	0/70	MD
Bataller et al. [51]	58	46/58	12	5/58	1/58	4/58	3/58	0/58	3/43
Baines et al. [52]	660	437/453	51	78/660	35/660	4/660	5/660	0/660	MD
Campagna et al. [53]	217	213/217	12	4/217	0/217	1/217	3/217	0/217	2/217
Kalis et al. [54]	128	97/114	12	MD	MD	MD	2/114	0/114	MD
Van den Akker et al. [55]	124	62/118	36	17/124	3/124	3/124	4/124	0/124	1/61
Ferrando et al. [56]	44	35/44	24	2/44	2/44	0/44	0/44	0/44	0/44
Lamblin et al. [57]	30	22/30	36	3/30	3/30	0/30	1/30	0/30	MD
Lallemant et al. [58]	160	138/160	33	8/160	2/160	2/160	1/160	1/160	MD
Total	5,818	5,467/5,936 (92.1%)		265/5,182 (5.1%)	147/5,037 (2.9%)	110/5,151 (2.1%)	116/5,820 (2.0%)	44/4,895 (0.9%)	45/1,898 (2.3%)

All mesh monofilament polypropylene except for PR and PPS

*MD* missing data, *PE* polyester, *PPS* monofilament polypropylene-dimethyl siloxane (silicone)

From the International Continence Society 7th edition International Consultation on Incontinence textbook (2023) [12]. Included with the authors’ permission

Recently, van Oudheusden et al. reported on a Dutch RCT (*n* = 64) comparing SCP with SSLF for the management of vault prolapse [59]. The RCT ceased early and was significantly underpowered to detect a difference between the groups. The authors then added 115 prospectively recruited non-randomised patients undergoing the same interventions. The 12-month attrition rate in this cohort was high, at 38% (44 out of 115). In addition, the primary outcome was unconventionally the disease-specific quality-of-life questionnaires, including the Urinary Distress Inventory, the Defecatory Distress Inventory, and the Incontinence Impact Questionnaire, and no differences in these outcomes were reported at 12 months. A secondary outcome measure defined as no pelvic organ prolapse beyond the hymen, absence of bothersome bulge symptoms, and no surgical or pessary re-treatment also failed to identify any differences between the interventions. Given the underpowered nature of this trial, along with the lack of randomisation of patients and high attrition rate, there are significant concerns regarding both the risk of bias and the clinical applicability of these findings.

Zhang et al. reported a meta-analysis of five RCTs and eight non-randomised trials examining SCP outcomes compared with SSLF that included 4,120 women [60]. The review found that SSLF was associated with a lower anatomical success rate than SCP (88.32% and 91.45% respectively; OR 0.52, 95% CI 0.29–0.95, *p* = 0.03) and higher recurrence (11.58% and 8.32%; OR 1.97, 95% CI 1.04–3.46, *p* = 0.04). Although the post-operative follow-up time for patients was adequate, with all but two of the studies included offering at least 24 months’ follow-up, the study is limited by several anomalies. The inclusion of nonrandomised and retrospective trials detrimentally impacts the quality of evidence. In addition, amongst the trials included there was significant heterogeneity, particularly in the definitions individual studies used to classify a successful anatomical repair. These factors may introduce bias and thus although SCP is shown to have favourable outcomes in this review, there remains ongoing concern about the quality of the presented evidence.

Overall, the evidence base strongly supports SCP as the gold standard for the surgical repair of apical vaginal prolapse. This is based on superior rates of anatomical success, decreased patient awareness of prolapse, and a lower need for repeat surgeries.

### Quality-of-Life Outcomes

Guan and Huan published a systematic review assessing quality-of-life improvements amongst patients undergoing various pelvic organ prolapse surgeries [61]. The review included 49 trials and compared SCP (*n* = 945) with SSLF (*n* = 713) and USLS (*n* = 400). The post-operative follow-up period ranged from 1 to 84 months and examined patient outcomes via the Pelvic Floor Distress Inventory–20 (prolapse, urinary, and bowel symptoms, with the higher the number, the greater the dysfunction). The weighted mean improvement rate (pre- and post-operatively) was 75.34% ± 17.24% for SCP, 65.78% ± 8.24% for USLS, and 58.72% ± 12.13% for SSLF. A limitation of this study was the significant heterogeneity in participant populations, surgical methods used, evaluation indexes, and follow-up times. With this in mind, SCP had superior patient-reported outcomes using a disease-specific questionnaire.

Pacquée et al. conducted a cohort study of 331 prospectively recruited patients who underwent a laparoscopic SCP for symptomatic prolapse (Pelvic Organ Prolapse Quantification [POP-Q] stage 2 or greater) [62]. As one of the primary outcomes, the trial used the Patient Global Impression of Change (PGI-C) score to quantify post-operative patient-reported outcomes. With a median follow-up period of 85.5 months, 84.4% (228 out of 270) patients reported a subjective improvement in quality of life post-operatively (PGI-C score of 4 or greater). In addition, only 2 out of 270 patients (0.7%) reported ongoing persistent bothersome prolapse symptoms at the end of their follow-up period. The strengths of this study include a long follow-up period, a significant number of participants, and a low attrition rate. The findings are limited with the descriptive nature of the cohort study being conducted in the absence of a comparative randomised group.

Patient-reported quality-of-life outcome measures stand as a pertinent cornerstone in the favourability of a surgical intervention. SCP has established favourable outcomes in this regard, with an overall high patient satisfaction and functional improvement in patient quality of life.

### Complications

The risk profile of SCP is expected to differ from vaginal surgical approaches to apical repair given the use of mesh and logistics of intra-peritoneal surgery. The complications of mesh are discussed separately in the uterine preservation and suturing material chapters below. Historically, the complications of SCP have been well documented in a 2015 meta-analysis [63] that included 79 studies. The review found that when compared with vaginal native tissue repairs, SCP was associated with a higher rate of small bowel obstruction (2.7% vs 0.2%, *p* < 0.1), mesh or suture complications (4.2% vs 0.4%, *p* < 0.01) and thromboembolic disease (0.6% vs 0.1%, *p* = 0.03). In contrast and more recently, a meta-analysis by Zhang et al. found that amongst the 4,120 participants included there was no significant difference in haemorrhage rates (OR 0.46, 95% CI 0.19–1.10, *p* = 0.08), wound infection rates (OR 0.46, 95% CI 0.21–1.02, included = 0.06) and gastrointestinal complications (OR 0.59, 95% CI 0.28–1.02, *p* = 0.06) [60]. This may reflect a gradual increase in surgical proficiency amongst surgeons performing SCP over time. To back this up further, van Oudheusden et al. [59] also found no difference in surgical complications when comparing SCP with SSLF for 6 weeks post-operatively. These complications included bleeding, visceral organ injury, urinary tract infections and wound infection. Surgical proficiency comes from a high surgical volume, which consequentially results in lower complication rates [64]. Secondary to its favourable outcomes, SCP has become increasingly utilised for the management of apical vaginal prolapse worldwide [2]. This increase in surgical volume occurs in the context of increased surgeon exposure, training and proficiency. Thus, in the decades to come the complication rates of SCP are expected to decline further.

In a recent study, Malekzadeh et al. compared complication rates amongst patients under and over the age of 65 who were undergoing an SCP [65]. Amongst the 312 participants in this retrospective study, no statistically significant differences were found with regard to intra-operative or post-operative complications, including 30-day re-admission rates, blood loss, urinary tract infections and constipation. Age is an established risk factor for pelvic organ prolapse [66], thus a significant proportion of patients undergoing SCP are expected to be elderly. Although every surgery should be undertaken on a case-to-case basis, age in itself did not increase surgical complications for SCP. A limiting factor to this finding was the retrospective nature of the study not allowing for adequate randomisation, which may introduce bias.

Lavikainen et al. conducted a large systematic review examining the venous thromboembolism risk associated with laparoscopic SCP [67]. The review involved 12 observational trials with 22,934 participants and found a symptomatic VTE risk estimate of 0.6% after a laparoscopic SCP. This figure was adjusted for thromboprophylaxis use, follow-up time and a stratified VTE risk based on patient factors. This VTE risk did rise to 1.4% when isolated to patients undergoing an open SCP (12 studies with 6,411 patients). Although high patient numbers were included in this systematic review, the confounding factors are the significant variation in thromboprophylaxis regime used amongst surgeons and the review did not account for any anti-haemorrhagic prophylaxis that may have been administered intra-operatively .

### Sacrocolpopexy for Uterine Prolapse

Level one data demonstrate that SCP is the gold standard procedure for vaginal vault prolapse [12]. Following this, some investigators have reported on the utilisation of SCP for uterine prolapse, as a concomitant hysterectomy, sub-total hysterectomy or a sacrohysteropexy (SHP). The majority of evidence surrounding SCP has been conducted exclusively in the context of post-hysterectomy prolapse. A meta-analysis [12] involving nine comparative studies with a total of 815 women found a lower anatomical success rate of −6.3% (−12%, −1%), *p* = 0.01 and a higher re-operation rate of 5.4% (2%, 9%), *p*=0.001, with SHP compared with SCP with concomitant hysterectomy (Table 2). Although anatomical outcomes were favourable for SCP and concomitant total hysterectomy, significant concerns remain relating to high rates of mesh exposure with this intervention [12, 68].

**Table 2:** Sacrohysteropexy versus hysterectomy and sacrocolpopexy [12]

Reference	Study type and surgery	Review (months)	Success, *N* (%) < stage 2		Reoperation prolapse		Complications	Mesh exposure, *N* (%)	
			HP	Hysterectomy	HP	Hysterectomy		HP	Hysterectomy
Costantini et al. [69]	Prospective cohort, ASHP vs TAH/SCP	51	31/34 (91)^a^	35/38 (92)^a^	0/34 (0)	0/38 (0)	Hematoma: 2 vs 4; transfusion: 2 vs 2	0/34	3/38 (8)
Costantini et al. [70]	Prospective cohort, ASHP vs TAH/SCP	12	32/32 (100)^b^	36/36 (100)^b^	0/32 (0)	0/36 (0)	MD	MD	MD
Cvach et al. [71]	Retrospective/prospective cohort, ASHP vs TAH/SCP	17	15/18 (83)^c^	8/8 (100)^c^	1/18 (6)	0/8	Transfusion: 0 vs 2	0/16	3/9 (33)
Jeon et al. [72]	Retrospective cohort, ASH vs TAH/SCP	36	35/35 (100)	60/63 (95)	MD	MD	Abscess: 0 vs 2; ureteral obstruction: 0 vs 1; SBO: 0 vs 3	0/35	5/63 (8)
Bai et al. [73]	Retrospective cohort, ASHP vs TAH/SCP	12	10/10 (100)^d^	18/19 (95)^d^	MD	MD	Transfusion: 3 vs 5; wound dehiscence and closure: 0 vs 2	0/10	3/19 (16)
Costantini et al. [74]	Retrospective, ASHP vs TAH/SCP	32	7/7 (100)	8/9 (89)	MD	MD	DVT/PE: 2; femoral neuropathy: 1; incisional hernia: 2	0/7	0/9
Pan et al. [75]	Retrospective cohort, LSHP vs TLH/LSCP	33	47/65 (72)^e^	30/34 (88)^e^	10/66 (15)^f^	0/34 (0)^f^	None	0/65	0/34
Illiano et al. [76]	Prospective cohort, LSHP vs TLH/LSCP	65	47/54 (87)	77/82 (94)	0/54	0/82	Transfusions: 2 vs 5; hernia: 1 vs 1; hematoma: 0 vs 1; sigmoid stenosis: 1 vs 0	2/54	6/82
Gagyor et al. [77]	Retrospective cohort LSHP vs LSH/LSCerP (*n* = 195) and TLH/LSCP (*n* = 38)	12	30/38 (79)	215/233 (92) x/38	MD	MD	Transfusions: 1 vs 1; bladder injury: 2 vs 8; Dindo grade III: 0 vs 4	1/38	3/233 x/38
Total			254/293 (87%)	272/289 (94%)	11/204 (5.4%)	0/196		2/221 (0.9%)	20/254 (7.9%)
			−6.3% (−12, −1%) *p* = 0.01		5.4% (2%, 9%) *p* = 0.001			−7% (−11, −3%) *p* < 0.001	

*HP* hysteropexy, *ASHP* abdominal sacral hysteropexy, *TAH* total abdominal hysterectomy, *SCP* sacrocolpopexy, *LSHP* laparoscopic sacrohysteropexy, *TLH* total laparoscopic hysterectomy, *LSCP* laparoscopic sacrocolpopexy, *LSH* laparoscopic supracervical hysterectomy, *MD* missing data

^a^< Stage 2 plus apex < −6 cm

^b^Apex < −6 cm

^c^Composite: anatomical cure (no prolapse beyond the hymen) plus no bulge symptoms

^d^< Stage 1

^e^< Stage 1 and at least 3 cm above the hymen

^f^Reoperation prolapse or pessary use

From the International Continence Society 7th Edition of the International Consultation on Incontinence textbook (2023) [12]. Included with the authors’ permission

The ICI 7th Edition of the Incontinence textbook (2023) compiled the cumulative mesh exposure rates across 26 trials [12]. It was found that the mesh exposure was 3.5-fold higher when an SCP was undertaken with a concomitant total hysterectomy (7.3%, 95 out of 1,303) versus 2.1% (40 out of 1,919, *p* <0.0001; Table 3).

**Table 3:** Rate of mesh exposures at sacrocolpopexy with and without total and supracervical hysterectomy [12]

Reference	Follow-up months	SCP surgery	Mesh	Vault POP	SCP + total hysterectomy	SCP + supracervical hysterectomy	*p* value
Open SCP							
Jeon et al. [78]	36	Open	TEFLON, MARLEX (PP), PP	0/35	5/63		
Jeon et al. [72]	66	Open	TEFLON, MARLEX(PP)	0/31	4/26		
Cundiff et al. [79]	24	Open	Mersilene (PE), PP, GORETEX	8/239	12/83		
Wu et al. [80]	15	Open	GORETEX, MERSILENE, PP	10/212	7/101		
Costantini et al. [69]	51	Open	MARLEX (PP)	0/34	3/38		
Bai et al. [73]	12	Open	Synthetic mesh	0/20	3/19		
Bensinger et al. [81]	12	Open	PP	0/35	4/49	0/37	
Brizzolara and Pillai-Allen [82]	35	Open	80% PP, 20% allografts	0/64	1/60		
Culligan et al. [83]	24	Open	Synthetic mesh	3/234	3/11		
Cvach et al. [71]	17	Open	70% PP, 30% porcine	0/16	3/9 (33)		
Ginath et al. [84]	7	Open	PP	2/82		1/195	
Total for open SCP				23/1,002 (2.3%)	45/459 (9.8%)	1/232 (0.4%)	<0.0001
Laparoscopic SCP							
Stepanian et al. [23]	12	Laparoscopic	PP	2/272	3/130		
Tan-Kim et al. [85]	15	Laparoscopic ± RA	PP	5/110	13/57^a^	1/21	
Osmundsen et al. [86]	3	RA laparoscopic	PP		8/49	0/31	
Bojahr et al. [87]	8	Laparoscopic	PP	0/19	MD	0/151	
Warner et al. [88]	6	Laparoscopic	PP	1/95	9/187^a^	0/92	
Crane et al. [89]	2	RA laparoscopic	PP	6/118	3/79	0/33	
Myers et al. [90]	12	RA laparoscopic	PP		3/40	1/43	
Pan et al. [91]	33	Laparoscopic	PP	0/65	0/34		
Gracia et al. [92]	12	Laparoscopic	PP	0/15		0/30	
Nosti et al. [93]	9	Laparoscopic ± RA	PP		2/123^b^	1/59	
Davidson et al. [94]	6	Laparoscopic ± RA	PP		1/45^a^	2/116	
Illiano et al. [76]	65	Laparoscopic ± RA	PP	2/54	6/82		
van Zanten et al. [95]	48	RA laparoscopic	PP	1/34		2/61	
Campagna et al. [53]	12	Laparoscopic	Titanium-coated PP	1/59	2/18	0/131	
Culligan et al. [96]	66	RA laparoscopic	PP	0/76		0/240	
Total for laparoscopic SCP				18/917 (2.0%)	50/844 (5.9%)	7/1,008 (0.7%)	<0.0001
Total				40/1,919 (2.1%)	95/1,303 (7.3%)	8/1,240 (0.6%)	<0.0001

*PP* polypropylene, *PE* polyester, *RA* robotic-assisted, *SCP* sacrocolpopexy, *MD* missing data

^a^Some of these cases involve vaginal attachment of the mesh

^b^All of these cases involve vaginal attachment of the mesh

From the International Continence Society 7th Edition of the International Consultation on Incontinence textbook (2023) [12]. Included with the authors’ permission

Similarly, in a meta-analysis of 11 trials assessing mesh exposure risk at SCP, the risk of vaginal mesh exposure was significantly increased if a total hysterectomy was concomitantly performed (8.6%) to 2.2% (*p*<0.05), and if the SCP was done for a post-hysterectomy prolapse [97]. More recently, Matthews et al. followed up 182 previously randomised patients who underwent a laparoscopic or robotic SCP with a concomitant total hysterectomy [68]. The mean post-operative follow-up time was 3.9 years, and the mesh exposure rate was 7.7%. This mesh exposure rate is significantly higher than what has been established for SCP for vault prolapse [63]. Thus, the evidence establishes higher rates of mesh exposure associated with SCP and concomitant total hysterectomy. This is thought to be secondary to increased devascularisation of vaginal tissue (from the hysterectomy) causing tissue necrosis and eventually higher rates of mesh exposure [85]. Vaginal mesh exposure following SCP can have a significant impact on a patient’s quality of life and be particularly difficult to manage compared with vaginal mesh exposure following vaginal interventions [98]. Considering that mesh exposure rates are significantly increased when SCP is performed concomitantly with a total hysterectomy (compared with SCP for vault prolapse) the ICI has concluded that hysterectomy at the time of SCP should not be considered [12]. The issue is further complicated by the medico-legal intricacies associated with the use of pelvic mesh in modern urogynaecological practice. These risks need to be carefully explained to patients and close post-operative follow-up put in place.

A proposed method of minimising these higher rates of mesh exposure when performing SCP for uterine prolapse is by undertaking a supracervical hysterectomy (as opposed to a total hysterectomy). No RCTs have been conducted directly comparing a SCP alone (for vault prolapse) versus an SCP with concomitant supra-cervical hysterectomy. Glass Clark et al. conducted a retrospective cohort study that included 17,111 women who underwent either a robotic or a laparoscopic SCP with concomitant supra-cervical (*n* = 6,708, 38%) or total hysterectomy (*n* = 10,403, 61%) [99]. In addition to the discrepancy in participant numbers amongst the groups, there was also significant heterogeneity between the two arms. Women in the concomitant supracervical group were older (age 60 ± 11 vs 53 ± 13, *p* < 0.01) and less likely to be obese (4% vs 7%, *p* < 0.01). At the 2 years post-operative follow-up there was no difference in mesh exposure rates (supracervical *n* = 47, 0.7% vs total *n* = 65, 0.62%, *p* = 0.61). The issues with this retrospective review are namely that mesh exposure was determined through International Classification of Disease (ICD) coding. Thus, although mesh exposure rates are reportedly low in both arms, there may be significant under-reporting on documented ICD criteria (as opposed to prospective findings on clinical examination). In addition, the ICD coding was only for one centre; thus, hypothetically, if a patient had mesh exposure managed elsewhere, they were deemed to have had no mesh exposure in this review. Second, the follow-up period of 2 years is short considering that mesh exposure rates rise over time [63]. Finally, there was no documentation of the type of mesh used in the SCPs. Thus, although this retrospective review finds that SCP with concomitant supra-cervical hysterectomy versus total hysterectomy showed no difference in mesh exposure rates, caution should be applied when interpreting these results owing to concerns of both validity and risk of bias. Another, less frequently discussed, aspect of supra-cervical hysterectomy at SCP or SHP is its surgical management in the case of a recurrence prolapse (or surgical failure). Owing to difficulty in removing the cervix (after a supra-cervical hysterectomy at SCP) or a hysterectomy (after SHP) these procedures can be challenging, and the morbidity associated with them is not clearly described.

There are no RCTs directly comparing surgical outcomes of SCP versus SHP or SCP with a concomitant hysterectomy. Van Zanten et al. conducted an observational cohort study comparing anatomical cure rates of women with symptomatic prolapse ≥ stage 2 POP undergoing a robotic SCP (vault prolapse) or a robotic supracervical hysterectomy with sacrocervicopexy (RSHS) [100]. One hundred and eighty-eight patients in the robotic SCP arm and 117 patients in the RSHS arm were prospectively followed up for 12 months post-operatively. Although the apical success rate was similar (92% in the robotic SCP group and 99% for the RSHS group), the rate of anterior compartment prolapse was higher in the RSHS arm. This may be secondary to the cervix impeding the surgeon’s ability to adequately reduce a large anterior compartment prolapse [68]. Even though apical anatomical outcomes here are deemed comparable in the medium term, the primary issue is that there was no reporting on mesh exposure.

Selle et al. conducted a retrospective cohort study that reviewed complication rates from 4,194 patients who had undergone an SCP versus 2,878 patients who underwent an SCP and concomitant hysterectomy [101]. No difference in complication rates was observed between the two arms (OR 0.99, 95% CI 0.67–1.46, *p* = 0.963) when looking at blood transfusions, sepsis, wound infections and other medical/anaesthetic complications. The study only followed patients for 30 days post-operatively and no comment was made on visceral organ injury.

Nygaard et al. conducted a long-term follow-up of previously randomised patients who underwent the Colposcopy And urinary Reduction Efforts (CARE) trial [102]. The initial RCT recruited 233 continent women who were randomised to undergo an SCP with or without urethropexy for symptomatic POP. This review followed up 215 of those patients (92%) with a median follow-up time of 7 years. There was an acceptable attrition rate of 31% over the 7 years, with 126 out of 215 (59%) completing the 7-year follow-up. Using an estimated probability of failure, the study found an anatomical failure rate (anatomical success was defined as TVL + C > 2 cm OR one of the points Ba or Bp > +1 cm) of 24–48%. The estimated probability of mesh exposure was calculated using the Kaplan–Meier method and found to be 10.5% (95% CI 6.8, 16.1). Analysing the data further, 36% of patients underwent a concomitant hysterectomy at the time of SCP. In this group the rate of mesh exposure was 5 times higher. In addition, in 48% of patients permanent sutures were used to suture the mesh to the vagina and in >50% of patients a multi-filament mesh was used. These variables have all been associated with higher rates of mesh exposure [63] and may explain the unexpected and high rate of vaginal mesh exposure reported.

Two cohort studies have reported mesh exposure rates with the use of a monofilament polypropylene mesh with absorbable sutures with long-term outcomes [52, 100]. Van Zanten et al. conducted a prospective cohort study of robotic SCP (*n* = 144) using a monofilament polypropylene mesh with absorbable sutures [100]. Over a 12-month follow-up period, a low mesh exposure rate of 2.3% was found. Similarly, Baines et al. conducted a retrospective cohort study that found a mesh exposure rate of 0.7%, with a mean follow-up time of 4 years and 3 months when using a monofilament polypropylene mesh at SCP [52]. The trial initially started by using non-absorbable sutures to secure the monofilament polypropylene mesh to the vagina, but after an increased rate of mesh exposure, changed over to absorbable sutures.

### Permanent Versus Absorbable Sutures for Vaginal Attachment of Mesh

**Fig. 1:**
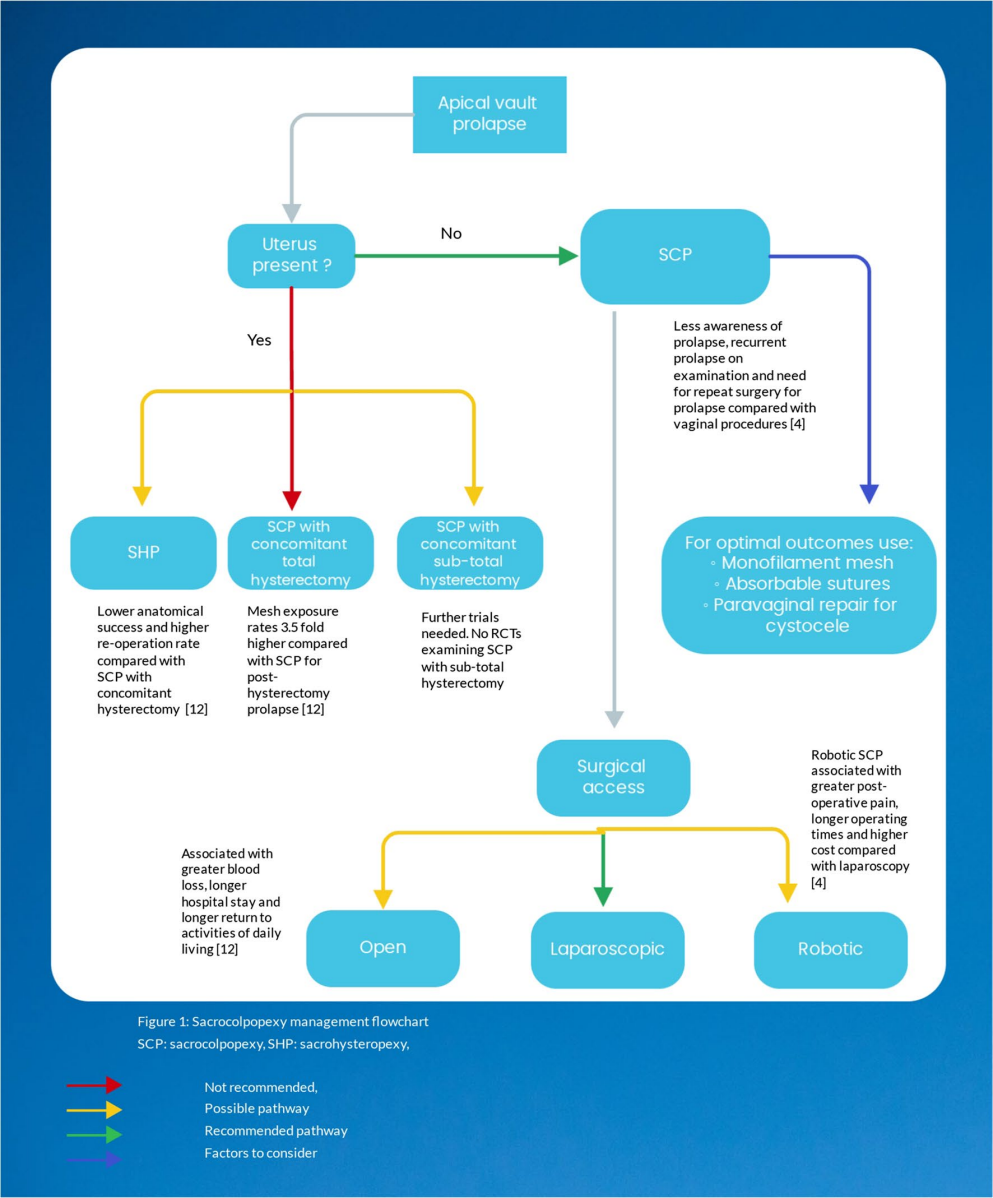
Sacrocolpopexy management flowchart. *RCT* randomised controlled trial, *SCP* sacrocolpopexy, *SHP* sacrohysteropexy

Two specific parameters define a surgeon’s choice of suture for SCP. First, the sutures’ effect on the anatomical recurrence of prolapse, and second, the sutures’ effect on the risk of complications, particularly mesh exposure. Chen et al. conducted a meta-analysis comparing absorbable versus non-absorbable sutures for SCP, involving four articles (two RCTs and two retrospective studies) with a total of 689 patients [103]. The duration of post-operative review ranged from 12 weeks to 12 months. The review found no difference in failure rates when using absorbable versus non-absorbable sutures for SCP (OR 0.75, 95% CI 0.34–1.66). There was, however, a lower rate of suture exposure in the absorbable suture arm (OR 0.18, 95% CI 0.06–0.58) and less of a need to remove the sutures (OR 0.14, 95% CI 0.03–0.61). Interestingly, there was no difference in mesh exposure rates between absorbable and non-absorbable sutures (OR 1.00, 95% CI 0.49–2.08). The main issue with this review is the short follow-up time for a complication such as mesh exposure, which is expected to increase over time. Therefore, with no established anatomical benefit and a higher rate of suture exposure, the use of permanent sutures for vaginal attachment of mesh at the time of SCP cannot be recommended (Fig. 1).

### SCP in the Management of Anterior Compartment Prolapse

A less frequently discussed aspect of SCP is its role in the management of a cystocele and concomitant surgeries performed at SCP. Lucot et al. conducted an RCT comparing outcomes of laparoscopic SCP (*n* = 130) versus transvaginal mesh (*n* = 132) for the treatment of primary stage ≥ 2 cystocele [104]. The multi-centre trial followed up patients for 12 months post-operatively and found in their intention-to-treat arm that exactly 46.5% of patients (59 out of 127) in both arms had no further prolapse. The anterior vaginal wall was documented as point Ba (POP-Q grading) and the mean pre- and post-operative difference was −1.3 (95% CI −1.6 to −1.1) in the SCP arm and −1.4 (95% CI −1.7 to −1.2) in the transvaginal mesh arm (*p* = 0.621). From a functional point of view, both groups reported favourable outcomes with 86.2% (118 out of 129) in the SCP arm and 81.6% (113 out of 128) in the transvaginal mesh arm, reporting an improvement (much better or very much better; *p* = 0.396). Similarly, De Castro et al. also assessed the role of SCP in the management of an anterior wall prolapse. The prospective RCT recruited 36 patients in the SCP arm versus 35 patients in the vaginal hysterectomy with SSLF and anterior vaginal mesh arm [105]. Thirty-four out of 36 patients (94.4%) in the SCP arm and 35 out of 35 patients (100%) in the other arm had either a stage 3 or 4 anterior compartment prolapse (POP-Q staging). The primary outcome was anatomical cure defined as Ba ≤ 0 (POP-Q grading) at the 12-month mark. The cure rate for point Ba in the SCP arm was 27 out of 36 (77.14%) and 25 out of 35 (73.53%) in the vaginal hysterectomy with SSLF and anterior mesh arm (*p* = 0.7277). Thus, this RCT showed that SCP produced good anatomical correction for large anterior wall prolapses compared with vaginal hysterectomy with SSLF and anterior vaginal mesh. The issues with the study revolve around a risk of bias given that it was a single-centre study with small numbers and all operations were performed by one surgeon. In addition, assessors were not blinded, and the SCPs were carried out as a sacrocervicopexy, with no comment on mesh exposure rates, which are expected to rise over time.

Brubaker et al. followed up 322 continent women previously randomised to receive either a Burch colposuspension or no Burch colposuspension (control) at the time of SCP [106]. At the 24-month follow-up there was a statistically significant reduction in point Ba (POP-Q system) from −1.8 ± 1.1 without a Burch colposuspension to −2.2 ± 0.9 with a Burch colposuspension (*p* < 0.001). In addition, at the 24-month follow-up patients in the concomitant Burch colposuspension arm had lower rates of SUI (32% versus 45% in the control group, *p* = 0.26) and lower rates of bothersome SUI (11.6% versus 25.2%, *p* = 0.04). Thus, SCP performed with a concomitant Burch colposuspension in continent women successfully reduced anterior compartment prolapses and symptomatic SUI over a 24-month follow-up period, compared with no Burch colposuspension at the time of SCP.

Given that a SCP anchors the vaginal vault to the sacral promontory posteriorly, it is often presumed that the procedure offers no support to the anterior compartment. These studies find that even large anterior compartment prolapses can be successfully repaired by an SCP if adequate anterior dissection and subsequent mesh placement is achieved. This is further enhanced if a paravaginal repair is performed at the time of SCP. In a systematic review of 13 RCTs, Costantini et al. found that retropubic interventions were performed in 87% and posterior colporrhaphy in 50% of the SCPs included [107]. These SCPs were analysed to establish superior outcomes in favour of SCP compared with vaginal interventions. These data suggest that SCP is rarely performed in isolation. Further RCTs are required in the specific context of SCPs for managing cystoceles, particularly considering the now rare use of transvaginal mesh for prolapse.

The ICI current evidence-based algorithm highlights native-tissue interventions for primary uterine prolapse and reserves SCP for post-hysterectomy prolapse [12]. Our practice is in keeping with these recommendations. Evidence regarding the efficacy of SCP has largely been conducted in the context of vault prolapse alone and has been extrapolated to include concomitant procedures. In addition, the mesh exposure rate is significantly higher if the SCP is done with a concomitant hysterectomy. Therefore, we recommend SCP for vault prolapse with absorbable sutures, a monofilament mesh and commonly performed with a paravaginal repair for optimal patient outcomes.

### Surgical Access (Open Versus Laparoscopic Versus Robotic)

The historical concerns of a laparoscopic approach to SCP (as opposed to an open SCP) have largely been debunked. The concerns centred around a proposed lower efficacy when an SCP was performed laparoscopically compared with an open approach. Costantini et al. undertook an RCT comparing surgical outcomes for women with a symptomatic prolapse stage ≥ 2 (POP-Q) who were randomised to either an abdominal (*n* = 60) or a laparoscopic (*n* = 61) approach to their SCP [37]. Cure was defined as prolapse stage 1 or less (POP-Q staging), point C/D (POP-Q system) −5 or less and total vaginal length of at least 7 cm. With a mean follow-up period of 41.7 months, the cure rate was 100% for both approaches. Therefore, the laparoscopic approach has comparable outcomes with an abdominal approach, with the additional benefits of less blood loss and a shorter hospital stay. The ICI (12) has implemented these findings by stating that a laparoscopic approach to SCP is associated with lower levels of blood loss, longer operating times, shorter hospital stays, quicker return to activities of daily living and a lower cost, with no difference in objective or subjective cure rates compared with an open approach (grade B evidence on the Oxford scale).

Given these favourable outcomes, the laparoscopic approach has been largely standardised. A recent survey [108] of 119 European gynaecologists found that 90.2% of respondents exclusively performed SCP laparoscopically, whereas the remaining 9.8% “sometimes” (i.e. not exclusively) used a robotic approach as well. The question now arises comparing laparoscopy with a robotic approach to SCP. De Gouveia De Sa et al. conducted a meta-analysis comparing the two modalities for SCP, which involved NICE trials and 1,157 participants [109]. No significant difference was found between laparoscopic and robotic approaches to SCP regarding anatomical outcomes, intra-operative complications (OR 0.78, 95% CI 0.42–1.43, *p* = 0.42), post-operative complications (OR 0.90, 95% CI 0.33–2.43, *p* = 0.83), mortality or hospital stay (MD −0.72, 95% CI 1.72–0.28, *p* = 0.16).

For many years proponents of the robotic approach argued that it was associated with a much quicker learning curve than the laparoscopic approach. Van Zanten et al. conducted a prospective cohort study examining the learning curve associated with robotic SCP [110]. Two experienced surgeons underwent analysis of their surgical proficiency when undertaking robotic SCP through their complication rates, which were established via a cumulative sum analysis. The trial found that surgical proficiency for robotic SCP was obtained at 78 cases for both surgeons. Similarly, Linder et al. assessed the learning curve associated with robotic SCP through a retrospective chart review of 145 cases [111]. Over 7 years the mean operating time dropped from 5.3 h to 3.6 h. A plateau of operating time was achieved after 60 robotic SCP cases. Surgical proficiency was identified as 55 robotic SCP cases (for intra-operative complications) and 84 cases when assessing both intra-operative and post-operative complications. Thus, the learning curve for robotic SCP remains as steep as that of the laparoscopic approach. The road towards surgical proficiency for gynaecologists in the robotic domain is further challenged by the need for proctors when starting and the limited availability of the robotic device in most centres.

In the aforementioned Cochrane review “Surgery for women with apical vaginal prolapse” (2023), a meta-analysis of four trials comparing laparoscopic SCP with robotic SCP involving 947 women was undertaken [4]. The review found that robotic SCP was associated with greater post-operative pain, longer operating times and a higher cost, with similar rates of anatomical success and complications. Thus, currently, the laparoscopic approach remains more common [108], with comparable anatomical and functional outcomes compared with a robotic SCP.

### Nerve-Sparing SCP

Recently, some authors have suggested a concern that surgical dissection at the time of SCP can result in de-innervation (particularly of branches of the inferior hypogastric nerve) resulting in de novo symptoms of bowel, bladder and sexual dysfunction. Christmann-Schmid et al. conducted a prospective cohort study of 137 women who underwent a laparoscopic SCP for apical prolapse > stage 2 (POP-Q staging) [112]. The study utilised a nerve-sparing technique involving superficial peritoneal dissection under continuous visualisation of the inferior hypogastric nerve in order not to transect it. The study did not have a comparative control group and after a 36-month post-operative follow-up period there was a statistically significant reduction when comparing pre-operative and post-operative overactive bladder symptoms (34.6% reduction, *p* < 0.001), stress urinary incontinence (34.4% reduction, *p* < 0.001) and voiding dysfunction (24.7% reduction, *p* < 0.001). The primary concern here is the lack of a control group, which means that outcomes cannot be attributed to SCP alone versus SCP performed with a nerve-sparing technique. In addition, 13% of patients underwent a concomitant procedure such as a transvaginal tape (*n* = 13), anterior colporrhaphy (*n* = 4) or paravaginal repair (*n* = 2), which could explain post-operative symptomatic improvements. With this in mind, the reduction in patient-reported overactive bladder symptoms through the Australian Pelvic Floor Questionnaire was significantly higher than what has been reported previously [11]. A point of note here is that the improvement of pre-existing symptoms post-operatively cannot be translated into a lower rate of de novo symptoms. A lack of standardisation of the nerve-sparing technique, along with limited visualisation of the inferior hypogastric nerve branches at the time of peritoneal dissection, makes this difficult to implement practically. In addition, underpowered studies performed in the absence of a control group, with findings potentially impacted by concomitant procedures, means that nerve sparing cannot be recommended. RCTs are needed to determine the statistical and clinical benefit of this technique.

## The Future—Outpatient SCP: Same-Day Discharge

**Fig. 2:**
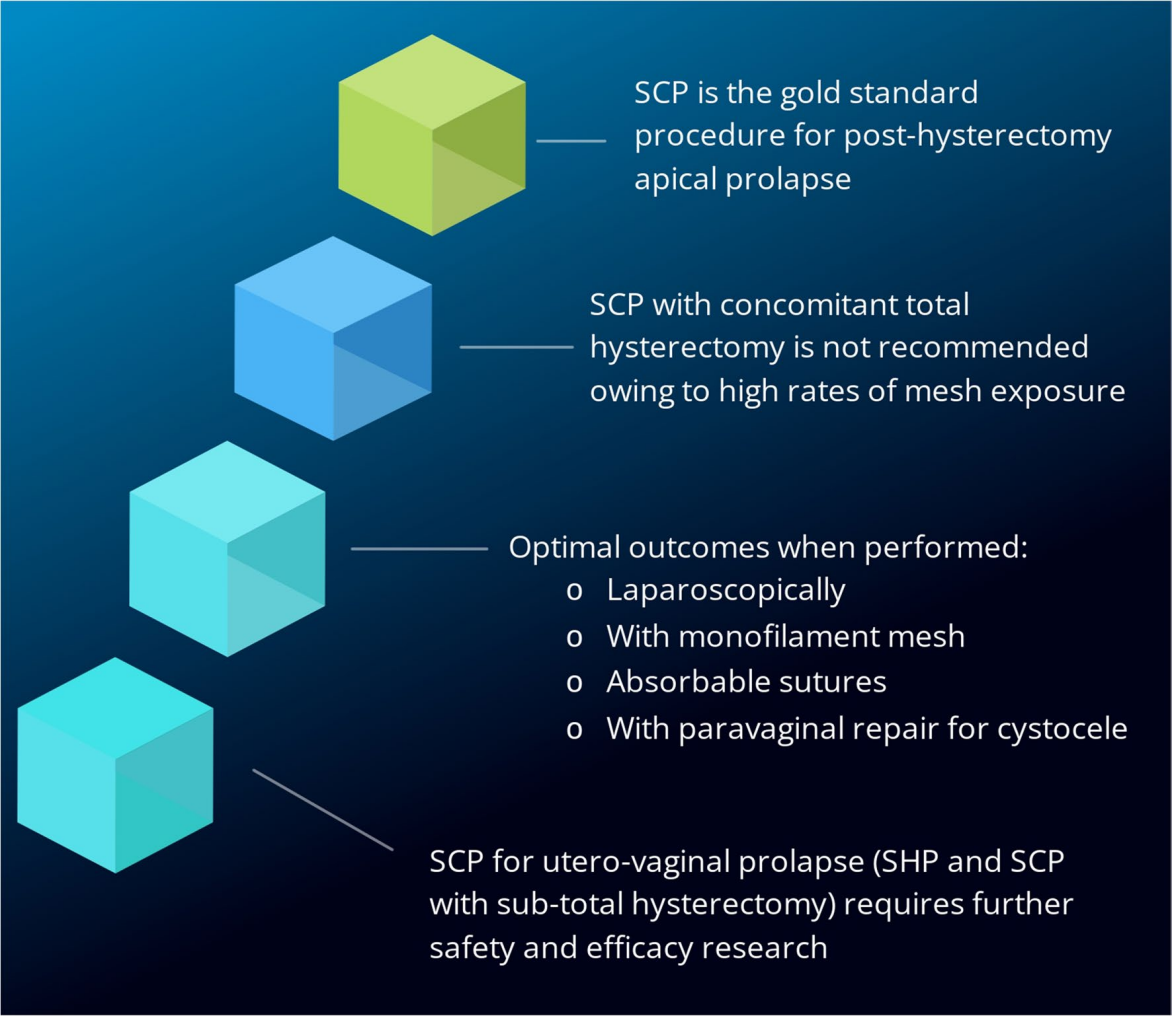
Conclusions. *SCP* sacrocolpopexy, *SHP* sacrohysteropexy

As a means of streamlining the logistics of patient care and increasing patient satisfaction, same-day discharge for SCP has been implemented in some centres. Guérin et al. conducted a retrospective review of 84 patients undergoing an SCP, either as an inpatient (*n* = 42) or an outpatient (*n* = 42) [113]. There was no difference in unscheduled visits (*p* = 0.58) or re-admission rate (*p* = 0.19) between the two groups. Patient satisfaction was gauged with a questionnaire asking patients if they were satisfied with their outpatient/inpatient care and they could answer on a five-point scale from very satisfied to dissatisfied. There was greater satisfaction amongst the inpatient cohort with 97.5%, stating that they were satisfied or very satisfied, whereas only 88.2% said the same in the outpatient group. Furthermore, 36% of patients in the outpatient group reported that they would have preferred to remain an inpatient post-operatively. Although this is a developing space, particularly with the possibility of remote (or virtual) care being provided to patients at home, given the acuity and unpredictability of post-operative complications, as things stand, inpatient management is safer and more accepted by patients (Fig. 2).

## Conclusion

Sacrocolpopexy with the use of monofilament mesh, absorbable sutures and commonly undertaken with a paravaginal repair remains the gold standard intervention for management of vaginal vault prolapse owing to its surgical durability, low complication rate and high patient satisfaction. The laparoscopic approach remains the preferred option in terms of low morbidity, quicker recovery and lower cost compared with alternative access options. Concomitant total hysterectomy at SCP is not recommended owing to the high rate of mesh exposure. The efficacy and safety of SCP with sub-total hysterectomy or hysteropexy has not been clearly established and requires further assessment through well-designed, rigorous RCTs.
